# Acute-Onset Panhypopituitarism Nearly Missed by Initial Cosyntropin Testing

**DOI:** 10.1155/2017/7931438

**Published:** 2017-10-03

**Authors:** Claudine A. Blum, Daniel Schneeberger, Matthias Lang, Janko Rakic, Marc Philippe Michot, Beat Müller

**Affiliations:** ^1^Medical University Clinic, Kantonsspital Aarau, Aarau, Switzerland; ^2^Department of Pneumology, Medical Clinic, Kantonsspital Baden, Baden, Switzerland

## Abstract

**Introduction:**

Diagnosis of adrenal crisis and panhypopituitarism in patients with septic shock is difficult but crucial for outcome.

**Case:**

A 66-year-old woman with metastasized breast cancer presented to the ED with respiratory insufficiency and septic shock after a 2-day history of the flu. After transfer to the ICU, corticosteroids were started in addition to antibiotics, as the patient was vasopressor-nonresponsive. Diabetes insipidus was diagnosed due to polyuria and treated with 4 mg desmopressin. Thereafter, norepinephrine could be tapered rapidly. On day 2, basal cortisol was 136 nmol/L with an increase to 579 nmol/L in low-dose cosyntropin testing. Polyuria had not developed again. Therefore, corticosteroids were stopped. On day 3, the patient developed again nausea, vomiting, and polyuria. Adrenal crisis and diabetes insipidus were postulated. Corticosteroids and desmopressin were restarted. Further testing confirmed panhypopituitarism. MRI showed a new sellar metastasis. After 2 weeks, stimulated cortisol in cosyntropin testing reached only 219 nmol/l, confirming adrenal insufficiency.

**Discussion:**

The time course showed that the adrenal glands took 2 weeks to atrophy after loss of pituitary ACTH secretion. Therefore, a misleading result of the cosyntropin test in the initial phase with low basal cortisol and allegedly normal response to exogenous ACTH may be seen. Cosyntropin testing in the critically ill should be interpreted with caution and in the corresponding clinical setting.

## 1. Introduction

We present the case of acute-onset panhypopituitarism due to a rapid growing sellar metastasis of breast cancer, where adrenal crisis and manifest diabetes insipidus were triggered by a lower respiratory tract infection.

## 2. Case Presentation

A 66-year-old woman presented to the emergency department with a two-day history of fever, cough, vomiting, and lethargy. Past medical history revealed a recently diagnosed recurrence of breast cancer with diffuse bone and mediastinal metastases. The patient was hypotensive (RR 85/55 mmHg, HR 105/min) and febrile (39.3°C). Arterial blood gas analysis showed global respiratory insufficiency. A chest computed tomography revealed an infiltrate in the left lower lobe. Fluid resuscitation and treatment with broad spectrum antibiotics were begun for presumed community-acquired pneumonia with septic shock. The patient was admitted to the ICU, where she required high doses of intravenous fluids and circulatory support with norepinephrine up to 0.4 ug/kgKG/min. Adjunct treatment with corticosteroids was started after obtaining a random cortisol of 258 nmol/L. The clinical condition of the patient stabilized rapidly, and norepinephrine was tapered within 8 hours after beginning of corticosteroid therapy. Confusingly, cosyntropin testing 14 hours later showed a basal cortisol of 136 nmol/L with an adequate absolute and relative (>250 nM) increase of cortisol after 30 minutes to 579 nmol/L.

The presence of polyuria (600–800 ml/hr) despite septic shock raised the suspicion of diabetes insipidus, which was confirmed with a serum osmolality of 320 mosm/kg and a urine osmolality of 148 mosm/kg. Consequently, four ug of desmopressin was administered intravenously.

After 36 hours, corticosteroid infusion was stopped, as the formal requirements for adrenal insufficiency and critical-illness-related corticosteroid insufficiency [1, 2] were only marginally met. Furthermore, as only one dose of desmopressin had been given and polyuria had not developed again, desmopressin was paused as well. The patient was then discharged from the ICU.

The following day, the patient complained of nausea, vomiting, and polyuria again. Vital signs were stable, but due to the clinical presentation, adrenal crisis was postulated and methylprednisolone 40 mg intravenously every 8 hours was administered. Furthermore, the patient failed to concentrate urine with a urine osmolality of 146 mmol/l and hypernatremia of 151 mmol/l. Manifest central diabetes insipidus was diagnosed and desmopressin treatment was restarted.

The diagnosis of acute septic shock due to influenza A/H1N1-pneumonia was made retrospectively due to positive nasopharyngeal swabs.

Further exploration of pituitary axis testing confirmed panhypopituitarism with a low FSH level in a postmenopausal patient and low serum levels of TSH, free T4, growth hormone, and IGF-1. Also, posterior lobe pituitary insufficiency was confirmed with levels of vasopressin and copeptin [3] in the low-normal range at the presence of high urine output with a low urine osmolality and high serum sodium (for detailed laboratory values, see Table 1).

**Figure 1:**
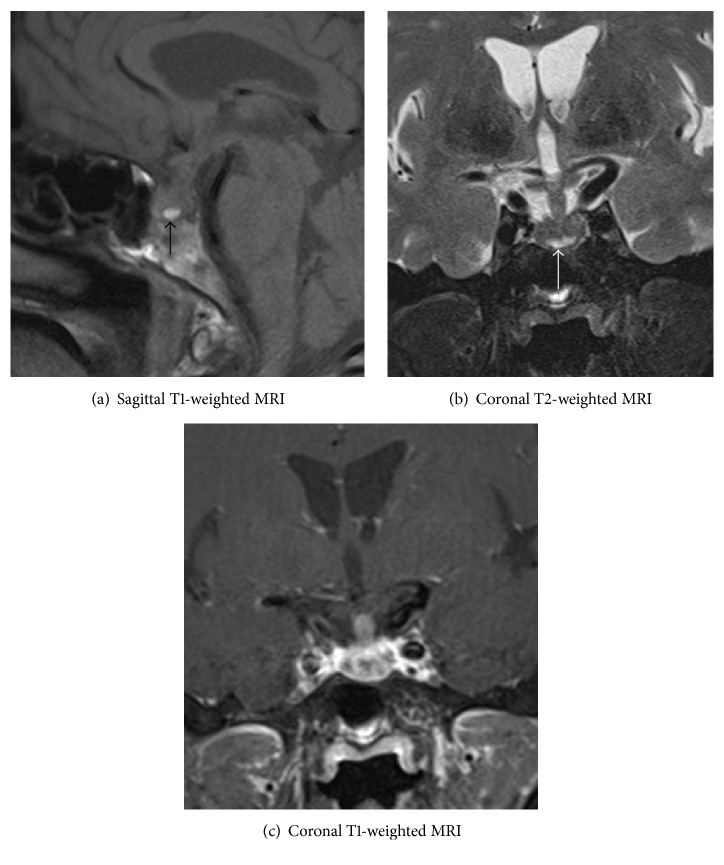
Inhomogeneity of the pituitary indicative of local metastases. Sagittal T1-weighted MRI (a) and coronal T2-weighted MRI (b) of the pituitary showed signal increase suggesting hemorrhage (arrows). Coronal T1-weighted MRI (c) with contrast shows inhomogeneous enhancement of pituitary with metastasis.

Pituitary MRI showed signal increase suggesting hemorrhage in sagittal T1-weighted and coronal T2-weighted MRI, but coronal T1-weighted MRI with contrast showed inhomogeneous enhancement of pituitary with metastasis. The concluding diagnosis was a sellar metastasis (Figure 1) of the known metastasizing breast cancer. This lesion was new, as it had not been present in a PET scan 2 months earlier.

After 2 weeks, stimulated cortisol levels in the repeated low-dose cosyntropin test reached only 219 nmol/l.

## 3. Discussion

Acute panhypopituitarism is rare and mostly due to acute ischemia or hemorrhage of the pituitary. In our patient, the reason for panhypopituitarism was a fast expanding sellar metastasis, with a pulmonary infection as a trigger for an adrenal crisis and manifest diabetes insipidus. The time course of the low-dose cosyntropin testing showed that it took two weeks for the adrenal glands to atrophy after loss of pituitary ACTH stimulation. Importantly, as exemplified in our patient, a misleading result of the cosyntropin testing in the initial phase with allegedly normal response to exogenous ACTH may be seen. The clinical presentation and suspicion in these patients are crucial together with the correct interpretation of an inadequately low random cortisol. Random serum cortisol and the response to cosyntropin test depend on the individual stress level [4, 5] and have to be interpreted with caution in critically ill patients with acute onset of a disease. Importantly, the critical care setting already represents a major stress stimulus for serum cortisol. Therefore, cosyntropin testing should not be routinely performed in the critical care setting [6, 7]. If adrenal failure in addition to septic shock or critical illness is suspected despite a borderline random cortisol, failure to stimulate after cosyntropin testing may help to diagnose primary adrenal insufficiency [8]. However, the present case shows that a high stimulated cortisol level does not rule out secondary adrenal insufficiency.

## Figures and Tables

**Table 1:** Laboratory values.

Variable	Reference range, adults	One week before admission	Day 1, 10 a.m.	Day 1, 10 p.m.	Day 2	Day 3	Day 4	Day 8	Day 15	Day 32
Hematocrit (%)	36.0–45.0	0.423	0.433	0.353	0.372	0.297				0.384
Hemoglobin (g/l)	120–155 g/l	140	137	107	118	97				118
White cell count (per mm^3^)	4000–10000	5700	9500	9400	6400	7000				7390
Thrombocytes (G/l)	140–400	234	287	277	292	243				306
Sodium (mmol/l)	136–146	148	139	153	147	147	151		143	144
Potassium (mmol/l)	3.6–5.0	3.6	3.9		3.8	4.3	4.2		4.3	4.3
Osmolality (mosmol/kg)	280–300		298	320			310		298	
Urine osmolality (mosmol/kg)	100–1200			148	274		146	271		
Urea (mmol/l)	2–7		8.1		4.6	4.9			5.7	7
Creatinine (umol/l)	53–94	61	170		120	91	83		90	55
Procalcitonin (ug/l)	<0.5	0.18	0.47		3.88					
C-reactive protein mg/l	<3.0	15.8	79.2		71.3	100	47.3			
Copeptin (pmol/l)	<31		4.8		2.3					
Vasopressin (ADH) (pmol/l)	1.0–4.2			<0.90						
TSH (mU/l)	0.4–4.0		0.14		0.05		0.07	0.1		
T3 (nmol/l)	0.90–2.60				2.05		0.91			
fT4 (nmol/l)	9.9–19.3						10.1	8.71		
HGH (ug/l)	<7				0.23					
IGF1 (ug/l)	59–195				64.7					105
FSH (U/l)	19–140				1.08					1.77
Prolactin (ug/l)	2.2–28						73.6			94.3
Morning cortisol (nmol/l)	140–700		258							
Cosyntropin test (low dose)										
Cortisol 0′ (nmol/l)				136				292	122	67
Cortisol 30′ (nmol/l)	>500			579				497	356	219
